# Prognostic Significance of Cyclin E1 Expression in Patients With Chordoma: A Clinicopathological and Immunohistochemical Study

**DOI:** 10.3389/fonc.2020.596330

**Published:** 2020-11-17

**Authors:** Ran Wei, Dylan C. Dean, Pichaya Thanindratarn, Francis J. Hornicek, Wei Guo, Zhenfeng Duan

**Affiliations:** ^1^Musculoskeletal Tumor Center, Beijing Key Laboratory of Musculoskeletal Tumor, Peking University People’s Hospital, Beijing, China; ^2^Sarcoma Biology Laboratory, Department of Orthopedic Surgery, David Geffen School of Medicine at UCLA, Los Angeles, CA, United States; ^3^Department of Orthopedic Surgery, Chulabhorn Hospital, HRH Princess Chulabhorn College of Medical Science, Bangkok, Thailand

**Keywords:** chordoma, cyclin E1, prognosis, biomarker, tissue microarray, immunohistochemical staining

## Abstract

**Purpose:**

Chordomas are rare, slow-growing sarcomas without any accepted prognostic biomarkers. Owing to their proximity to critical neurovascular structures, discovering predictive biomarkers in chordoma has been a significant research effort because it may potentially reduce risky therapies in patients with less aggressive tumors. In response, because cyclin E1 overexpression correlates with patient prognosis in several malignancies, we investigated its expression in chordoma and whether it informs patient prognosis.

**Methods:**

Seventy-five chordoma patient specimens were enrolled in a tissue microarray (TMA) to evaluate cyclin E1 expression *via* immunohistochemical staining. Western blot was used to assess cyclin E1 expression in chordoma cell lines and fresh tissues. We then correlated cyclin E1 staining intensity in the TMA to clinicopathological features and chordoma patient outcomes.

**Results:**

Sixty-three percent of the chordoma patient specimens in the TMA, fifty-six percent of the fresh chordoma tissues, and all chordoma cell lines showed high cyclin E1 expression. In TMA analysis, cyclin E1 expression positively correlated to chordoma patient disease status. By survival analysis, high cyclin E1 expression was an independent prognostic risk factor for chordoma patients along with advanced disease status and positive surgical margin.

**Conclusion:**

Cyclin E1 is a promising biomarker predicting chordoma patient prognosis.

## Introduction

Chordomas are rare bone sarcomas that arise from the transformed remnants of notochord, with an incidence of 0.1/100,000 people per year (1). It principally affects elderly patients at the skull base (41.1%), mobile spine (27.4%) and sacrum (31.5%) (2). Surgery is the cornerstone therapeutic modality for chordoma, with adequate surgical margins representing the primary clinical goal for a favorable prognosis (3–5). And although the 5-year survival rate for chordoma patients is an optimistic 70-81%, local recurrence and subsequent metastasis occurs approximately half the time after surgery, with no systemic therapies having shown major benefit (1, 5, 6). And while their diagnosis is made according to clinicopathological features and brachyury expression which is a prominent transcription factor in notochord development, brachyury is a diagnostic biomarker and therapeutic target rather than a prognostic biomarker (7–10). In short, there are no well-recognized biomarkers predictive of chordoma patient prognosis. For the patient, the lack of accurate predictive biomarkers can result in overtreatment of less aggressive chordomas *via* surgery or adjuvant radiation therapy. In addition, even with aggressive surgery, recurrent rates remain high. There is, therefore, an urgent need for the identification of chordoma biomarkers to better ascertain patient prognosis and whether high-risk surgeries or adjuvant therapies are indicated.

Cyclin E1 was originally described in 1991 as the prototype cyclin E. It is a 47 kDa protein localized within both the nucleus and cytoplasm (11). By complexing with cyclin-dependent kinase 2 (CDK2), it phosphorylates numerous downstream proteins such as Rb and is strong promoter of G1-S transition, a well-known tumorigenic step (12). Cyclin E1 overexpression has been investigated in more common malignancies, and has shown to correlate with therapeutic response and prognosis (13–18). Despite these established findings in other cancers, the expression and clinical significance of cyclin E1 in chordoma is unknown.

In response, we sought to investigate the expression of cyclin E1 in chordoma and its correlation, if any, to clinicopathologic features or prognosis within chordoma patients. To our knowledge, this is the first and only work evaluating cyclin E1 expression in chordoma and its clinical significance.

## Materials and Methods

### Ethics

The study protocol and the consent of the informed patients were approved by the Partners Human Research Committee (number: 2007-P-002464/5) of Partners HealthCare as previously described (19). All patients provided their written informed consent to participate in this study.

### Chordoma Tissue Samples and Tissue Microarray

Seventy-five chordoma patient specimens in formalin fixed paraffin embedded (FFPE) blocks obtained during surgery were used to construct the tissue microarray (TMA) as previously described (19). The inclusion criteria included: 1) patients with chordoma confirmed by histological diagnosis; 2) patients underwent surgical treatment; 3) patients with complete clinical and follow-up data. The exclusion criteria included: 1) peri-operative death; 2) patients with incomplete clinical and follow-up data. The demographic and clinical information of 75 patients was reviewed and documented from a prospective database. In the course of the TMA construction, three sites of each FFPE block were selected to assemble the recipient master block. Representative triplicate 0.5 mm diameter core biopsies of each tissue block were obtained through pathology reports and pathologist read hematoxylin and eosin (HE)-stained slides. In addition, nine fresh chordoma tissue samples were obtained for study.

### Immunohistochemistry Staining and Assessment

Immunohistochemistry (IHC) staining was performed to evaluate for cyclin E1 expression. In brief, the paraffin-embedded slide was baked for 1 h at 60°C before xylene deparaffinization. The slide was then rehydrated through graded ethanol (100% and 95%). We then used 3% hydrogen peroxide to quench endogenous peroxidase activity after heated epitope retrieval. After blocking for 1 h with normal goat serum, the slide was incubated with polyclonal rabbit antibody to human cyclin E1 (Cell Signaling Technology, MA, USA. Catalog# 20808S) overnight in a humidified chamber set at 4°C. Afterwards, the bound antibody was detected by SignalStain^®^ Boost Detection Reagent (Cell Signaling Technology, MA, USA) and SignalStain^®^ DAB (Cell Signaling Technology, MA, USA). Finally, all sections were counterstained with Hematoxylin QS (Vector Laboratories, CA, USA), and the slide was mounted with VectaMount AQ (Vector Laboratories, CA, USA) for long-term preservation.

**Figure 1:**
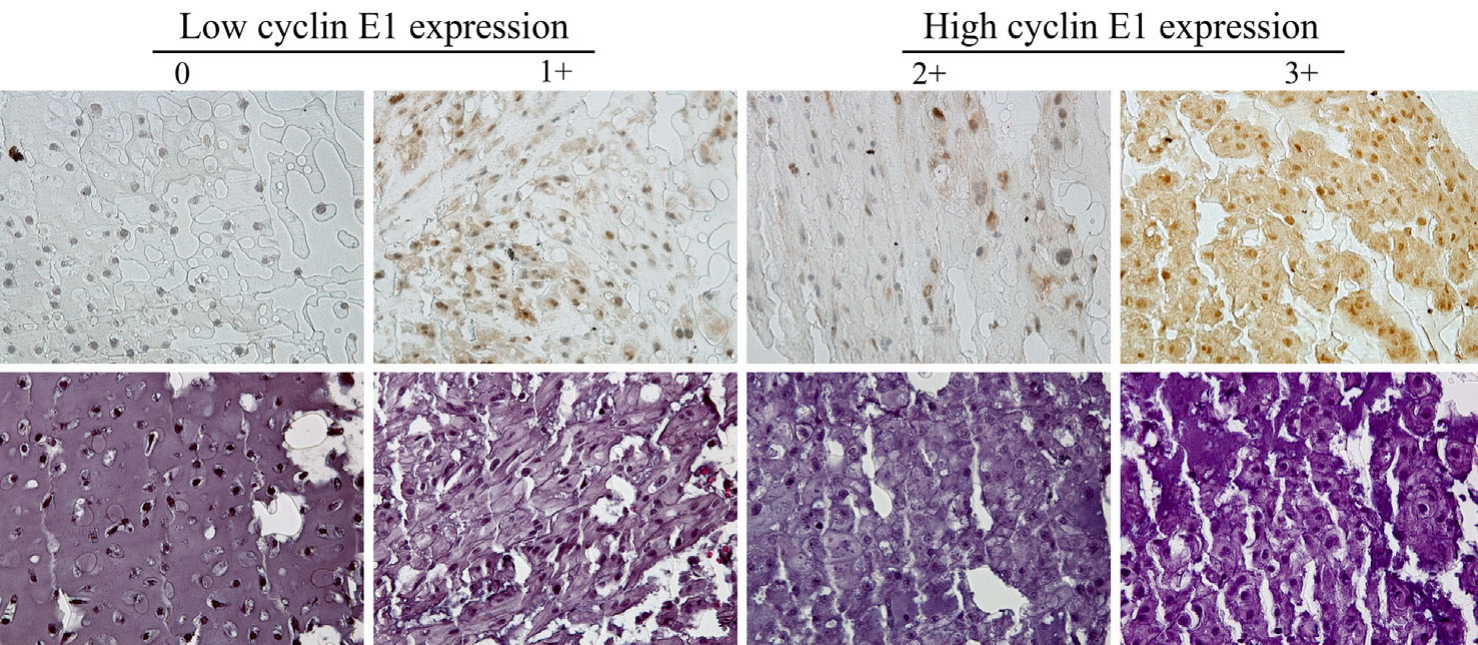
Assessment of cyclin E1 expression in the chordoma tissue microarray by immunohistochemistry. Different immunohistochemistry staining intensities of cyclin E1 and HE in chordoma tissues are shown in representative images. Staining patterns were divided into 4 groups: 0, no staining; 1+, weak staining; 2+, moderate staining; 3+, strong staining (Original magnification, 400×). Low and high cyclin E1 expression groups included chordoma specimens with the staining score of 0 to 1+ and 2+ to 3+, respectively.

Two independent pathologists blinded to patient clinical information and tumor characteristics assessed and scored the IHC-stained slide. Cyclin E1 expression was scored and categorized according to the staining intensity of chordoma tissues: 0, no staining; 1+, weak staining; 2+, moderate staining; 3+, strong staining. The low cyclin E1 expression subset included group 0 and 1+, while the high cyclin E1 expression subset included group 2+ and 3+ (**Figure 1**). A Nikon Eclipse Ti-U fluorescence microscope (Diagnostic Instruments Inc., MI, USA) with a SPOT RT™ digital camera (Diagnostic Instruments Inc., MI, USA) was used to obtain cyclin E1 staining images.

### Human Chordoma Cell Lines and Cell Culture

The human chordoma cell line UCH2 was established and kindly provided by Dr. Silke Brüderlein (University Hospitals of Ulm, Ulm, Germany) (20). The cell line CH22 was established in our laboratory as previously reported (21). The UCH2 and CH22 cells were incubated at 37°C with 5% CO_2_ in Dulbecco’s Modified Eagle Medium (DMEM) (Life Technologies Corp., NY, USA) supplemented with 10% fetal bovine serum (Sigma-Aldrich, MO, USA) and 1% penicillin/streptomycin (Life Technologies, CA, USA).

### Protein Preparation and Western Blot

We used 1× RIPA lysis buffer (Sigma-Aldrich, MO, USA) combined with protease inhibitor cocktail tablets (Roche Applied Science, IN, USA) to extract protein from cells and fresh tissues. The DC™ protein assay reagents (Bio-Rad, CA, USA) and a spectrophotometer SPECTRA max 340PC (Molecular Devices, LLC., CA, USA) were then used to determine the protein lysate concentrations. Briefly, our Western blot protocol began with an SDSPAGE gel to run the denatured proteins before transfer to nitrocellulose membranes. These membranes were incubated with monoclonal rabbit antibodies to human cyclin E1 (1:1,000 dilution, Cell Signaling Technology, MA, USA) and mouse monoclonal antibody for human β-actin (1:20,000 dilution, Sigma-Aldrich, MO, USA) at 4°C overnight after they were blocked in Odyssey Blocking Buffer (Li-COR Biosciences, NE, USA) for 1 h. Following incubation with the primary antibody, TBST was used as a membrane wash (4 times for 5 min each at room temperature). Next, goat anti-rabbit IRDye 800CW (926–32,211, 1:5,000 dilution) and goat anti-mouse IRDye 680LT secondary antibody (926–68,020, 1:15,000 dilution) (Li-COR Biosciences, NE, USA) were applied for 1 h at room temperature followed by another TBST membrane wash (four times for 5 min each at room temperature). Bands were detected using Odyssey Infrared Fluorescent Western Blot Imaging System from Li-COR Bioscience (NE, USA), and Odyssey software 3.0 was used to quantify the bands.

### Statistical Analysis

GraphPad Prism 7 software (GraphPad Software, CA, USA) and SPSS 19.0 software (IBM Corp., Armonk, New York) were used for statistical analyses. Nonparametric testing (Mann-Whitney U test) was performed to compare the two groups and determine statistical significance. One-way analysis of variance and the least-significant difference test were performed for multiple comparisons. As for the survival analysis, overall survival (OS) was defined as the time from surgery (when the tissue was obtained) to death of the patient. Recurrence-free survival (RFS) was defined as the time from the surgery to the first local recurrence after this surgery. Metastasis-free survival (MFS) was defined as the time from surgery to the detection of new onset metastasis. The survival curves were produced by Kaplan-Meier methods. The relation between various key factors and OS, RFS, or MFS was evaluated by Cox regression both univariately and multivariately. Only those factors that were statistically significant (*P*<0.05) in the univariate analysis were involved in the multivariate analysis. A *P*-value < 0.05 was considered significant.

## Results

### Clinicopathological Characteristics of 75 Chordoma Patients Enrolled in TMA

The demographic and clinicopathological characteristics of 75 patients are shown in **Table 1**. There were 53 (70.7%) males and 22 (29.3%) females, with an average age of 59.5 ± 13.7 years. Thirty-one patients (41.3%) were treated for primary localized lesions while 44 patients (58.7%) had advanced disease, including 39 patients treated for recurrent lesions and 5 patients with metastasis. The majority of the lesions were located at sacrum (44, 58.7%). Surgeries were performed through an anterior and posterior approach in most patients (47, 62.7%), and negative surgical margins were completed in 18 patients (24.0%). Neoadjuvant radiation was performed in 57 patients (76.0%) without detailed information regarding the type and dose of the radiation.

**Table 1:** Demographic and clinical information of 75 chordoma patients enrolled in a tissue microarray.

Variables		N (%) or mean ± SD
Age		59.5 ± 13.7 years
Sex	Male	53 (70.7)
	Female	22 (29.3)
Location of lesion	Sacrum	44 (58.7)
	Mobile spine	31 (41.3)
Disease status	Primary	31 (41.3)
	Advanced	44 (58.7)
	Recurrent	39 (52.0)
	Metastatic	5 (6.7)
Surgical approach	Posterior only	28 (37.3)
	Anterior and posterior	47 (62.7)
Margin	Negative	18 (24.0)
	Positive	57 (76.0)
Neoadjuvant radiation	Received	57 (76.0)
	Not received	18 (24.0)
Follow-up time		77.5 ± 54.2 months

### Cyclin E1 Is Highly Expressed in Chordoma

**Figure 2:**
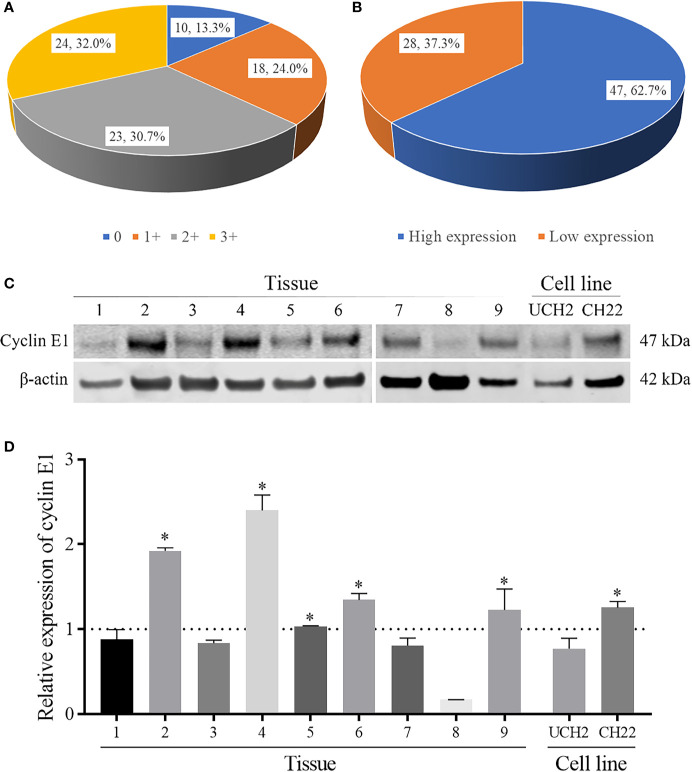
The expression levels of cyclin E1 in chordoma. **(A, B)** Pie chart showing frequency and percentage of different cyclin E1 expression levels and high/low cyclin E1 expression groups in chordoma tissue microarrays. **(C)** Western blots showing cyclin E1 expression in nine chordoma fresh tissues and cell lines (UCH2 and CH22). **(D)** Densitometry quantification of the Western blots of cyclin E1 from **Figure 2C**, presented as relative to β-actin expression. The data are presented in as mean ± SD of the experiment carried out in triplicate. * means the expression of cyclin E1 is stronger than the β-actin, indicating high cyclin E1 expression.

Cyclin E1 expression was first evaluated in TMA by IHC staining. Among 75 specimens, no staining was found in 10 specimens (13.3%) with the remaining 65 specimens having various staining intensities, including 1+ staining in 18 specimens (24.0%), 2+ staining in 23 specimens (30.7%), and 3+ staining in 24 specimens (32.0%) (**Figure 2A**). According to the aforementioned categorization criteria, high and low cyclin E1 expression was found in 47 (62.7%) and 28 (37.3%) specimens, respectively (**Figure 2B**).

We further evaluated cyclin E1 expression in nine fresh chordoma tissues and two chordoma cell lines. All chordoma tissue samples showed cyclin E1 expression, including five with high expression (55.6%) and 4 with low expression (44.4%). In chordoma cell lines, Western blots showed cyclin E1 was expressed in both UCH2 and CH22, with CH22 having notably high expression. (**Figures 2C, D**)

### Cyclin E1 Expression Correlated With Disease Status of Chordoma Patients

**Figure 3:**
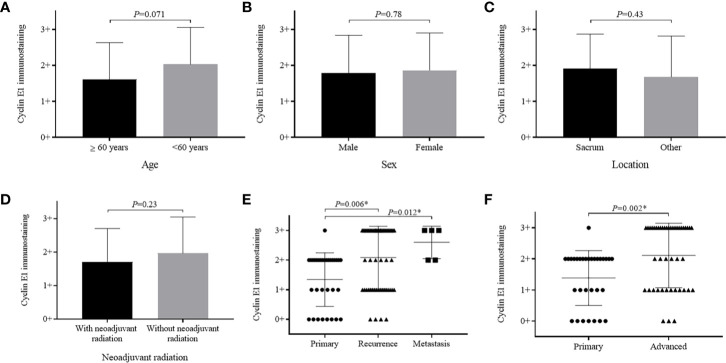
Relationship between cyclin E1 expression and clinicopathological characteristics of chordoma patients. Graphics illustrate no significant differences in cyclin E1 expression level among patients based on **(A)** age group, **(B)** sex, **(C)** tumor location or **(D)** with and without neoadjuvant radiation. Significant differences were observed in cyclin E1 expression based on patient disease status **(E, F)**. *Statistical significance.

Cyclin E1 expression did not significantly vary based on patient age, sex, or lesion location (**Figures 3A–C**). When patients with and without neoadjuvant radiation were compared, cyclin E1 expression was weaker in radiation-treated patients versus those without radiation, but did not meet statistical significance (1.7 ± 1.0 versus 2.0 ± 1.1, *P*=0.23) (**Figure 3D**).

Importantly, patients who had recurrent and metastatic chordomas prior to surgery showed significantly higher cyclin E1 expression compared to those patients with primary lesions (recurrence versus primary, 2.1 ± 1.1 versus 1.4 ± 0.9, *P*=0.006; metastasis versus primary, 2.6 ± 0.5 versus 1.4 ± 0.9, *P*=0.012) (**Figure 3E**). Essentially, a more advanced chordoma staging correlated to higher cyclin E1 expression (advanced versus primary, 2.1 ± 1.0 versus 1.4 ± 0.9, *P*=0.002) (**Figure 3F**).

### Cyclin E1 Overexpression Is an Independent Prognostic Factor in Chordoma

**Figure 4:**
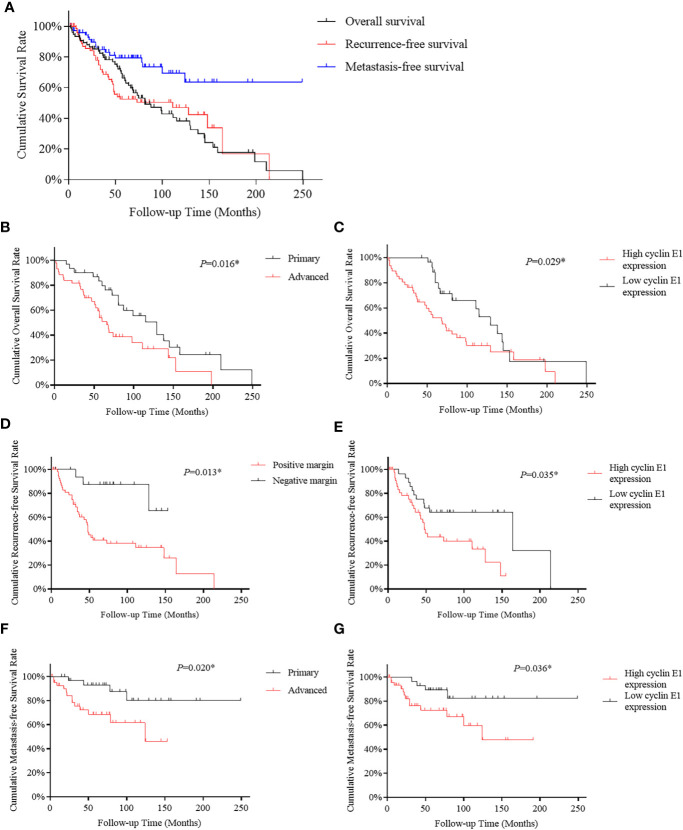
Kaplan-Meier survival curve derived results of survival analysis. **(A)** Overall, recurrence-free and metastasis-free survival curves of 75 chordoma patients. **(B, C)** Advanced disease status and high cyclin E1 expression were independent risk factors for overall survival in 75 chordoma patients. **(D, E)** Positive surgical margins and high cyclin E1 expression were independent risk factors for recurrence-free survival in 75 chordoma patients. **(F, G)** Advanced disease status and high cyclin E1 expression were independent risk factors for metastasis-free survival in 75 chordoma patients. *Statistical significance.

To identify whether cyclin E1 expression correlates to the prognosis of chordoma patients, survival analysis was performed on OS, RFS, and MFS of the 75 chordoma patients. In summary, after the tissue samples were obtained post-surgery, death occurred in 49 patients (65.3%), local recurrence was found in 37 patients (49.3%) and new onset metastasis was found in 17 patients (22.7%). Of the 75 chordoma patients, the mean OS in months was 102.3 (95% CI 83.7 to 121.0), RFS was 102.4 (95% CI 79.5 to 125.3) and MFS was 180.2 (95% CI 151.5 to 208.9). Five-year rates included OS of 63.1% (95% CI 51.7% to 74.5%), RFS was 52.4% (95% CI 40.2% to 64.6%), and MFS rate was 79.3% (95% CI 69.1% to 89.5%). Finally, the ten-year rates included OS of 38.2% (95% CI 25.3% to 51.1%), RFS of 47.0% (95% CI 33.9% to 60.1%), and MFS of 69.5% (95% CI 55.6% to 83.4%) (**Figure 4A**).

For OS, both univariate and multivariate analysis suggested advanced disease status and high cyclin E1 expression were independent risk factors (**Table 2**) (**Figures 4B, C**). For RFS, positive surgical margins and high cyclin E1 expression were identified by univariate and multivariate analysis to be independent risk factors (**Table 3**) (**Figures 4D, E**). And for MFS, advanced disease status and high cyclin E1 expression were independent risk factors (**Table 4**) (**Figures 4F, G**). Overall, cyclin E1 overexpression was an independent prognostic risk factor for poor chordoma patient outcomes.

**Table 2:** Univariate and multivariate analysis of overall survival for 75 chordoma patients.

Variables	Univariate			Multivariate		
	HR	95% CI	*P*	HR	95% CI	*P*
Age ≥60 yrs <60 yrs	0.73	0.40 to 1.31	0.28			
Sex Male Female	1.41	0.76 to 2.62	0.28			
Location Sacrum Other	1.01	0.56 to 1.81	0.99			
Disease status Primary Advanced	2.04	1.12 to 3.74	0.020*	2.11	1.15 to 3.86	0.016*
Surgical margin Positive Negative	0.69	0.34 to 1.39	0.30			
Neoadjuvant radiation With Without	0.63	0.36 to 1.12	0.12			
Cyclin E1 expression High Low	0.52	0.28 to 0.96	0.037*	0.50	0.27 to 0.93	0.029*

*Statistical significance; HR, hazard ratio; 95% CI, 95% confidence interval; yrs, years.

**Table 3:** Univariate and multivariate analysis for recurrence-free survival of 75 chordoma patients.

Variables	Univariate			Multivariate		
	HR	95% CI	*P*	HR	95% CI	*P*
Age ≥60 yrs <60 yrs	1.54	0.79 to 2.98	0.20			
Sex Male Female	1.90	0.94 to 3.81	0.072			
Location Sacrum Other	1.64	0.85 to 3.16	0.14			
Disease status Primary Advanced	1.67	0.84 to 3.33	0.14			
Surgical margin Positive Negative	0.20	0.062 to 0.67	0.009*	0.22	0.067 to 0.73	0.013*
Neoadjuvant radiation With Without	0.94	0.48 to 1.85	0.87			
Cyclin E1 expression High Low	0.41	0.20 to 0.86	0.018*	0.45	0.22 to 0.95	0.035*

*Statistical significance; HR, hazard ratio; 95% CI, 95% confidence interval; yrs, years.

**Table 4:** Univariate and multivariate analysis for metastasis-free survival of 75 chordoma patients.

Variables	Univariate			Multivariate		
	HR	95% CI	*P*	HR	95% CI	*P*
Age ≥60 yrs <60 yrs	1.73	0.66 to 4.54	0.27			
Sex Male Female	1.51	0.56 to 4.10	0.42			
Location Sacrum Other	0.81	0.30 to 2.19	0.68			
Disease status Primary Advanced	3.66	1.18 to 11.34	0.025*	3.84	1.24 to 11.92	0.020*
Surgical margin Positive Negative	0.33	0.075 to 1.43	0.14			
Neoadjuvant radiation With Without	1.28	0.47 to 3.45	0.63			
Cyclin E1 expression High Low	0.32	0.10 to 0.97	0.045*	0.30	0.097 to 0.93	0.036*

*Statistical significance; HR, hazard ratio; 95% CI, 95% confidence interval; yrs, years.

## Discussion

Overexpression of cyclin E1 has been recognized in various malignancies including lung cancer (51%–62%), breast cancer (53%–61%), liver cancer (36%–68%) and some sarcomas (29%–61%) (15, 17, 22–28). This elevated cyclin E1 expression has been attributed to gene amplification, transcription upregulation, and disrupted degradation. In terms of frequency, the cyclin E1 gene is one of the most commonly amplified genes in ovarian cancer (19%), anaplastic thyroid cancer (29%) and osteosarcoma (27-33%) (14, 17, 18, 29). Overexpression of transcription factors such as c-MYC and MEIS2 directly up-regulate cyclin E1 expression (30, 31). In addition, inactivation of core units central to the ubiquitin protein ligase complex lead to cyclin E1 overexpression, as this is its principal degradative mechanism (32). In our study, overexpression of cyclin E1 occurred in 62.7% of chordoma patients, and is consistent with findings in more common cancers. In total, our work supports cyclin E1 as a diagnostic biomarker in chordoma.

Our TMA analysis showed cyclin E1 expression positively correlates with chordoma patient disease status. Of note, these results echo works in other malignancies where high cyclin E1 expression is associated with high-staging as well as tumor aggression and therapeutic-resistance (28, 33, 34). Functionally, it is proposed that these carcinogenic features are in part due to the cyclin E1 role in shortening mitosis and facilitating chromosome instability, both of which are known to incite tumorigenesis (35, 36). It therefore follows and is clear that cyclin E1 overexpression advances chordoma.

Cyclin E1 predicts poor prognosis in several malignancies (15, 28, 37). In our study, cyclin E1 showed prognostic significance with respect to OS, RFS and MFS in chordoma patients. Because recurrence and subsequent metastasis are of particular significance in determining chordoma outcomes, the ability of cyclin E1 overexpression to function as a prognostic biomarker is especially noteworthy for correlating with RFS and MFS in our study (4). As a specific prognostic biomarker rather than pure diagnostic biomarker, cyclin E1 has the potential to inform clinical decision-making and therapy selection based on predicted progression and recurrence. This is especially important clinical information, as chordoma surgery and radiation therapy carry significant risks to the patient owing to its anatomic proximity to vital neurovascular structures. Taken together, cyclin E1 expression is a promising prognostic biomarker for chordoma patients that may help guide clinical decision making.

Finally, we assessed the predictive potential of other clinicopathological features for chordoma patients. As expected, an advanced disease status of chordomas engenders more aggressive future tumor behavior. It is an independent risk factor for OS and MFS in chordoma, which is similar to the results of previous studies (2, 5, 38). Moreover, we found a contaminated surgical margin to be an independent risk factor for RFS in chordoma patients. This is consistent to previous studies, and further suggests that an adequate surgical margin is imperative to optimize chordoma treatment and to avoid local progression and recurrence (1, 5). Furthermore, we found no correlation between neoadjuvant radiation with prognosis/cyclin E1 expression level. While the therapeutic efficacy of radiation for chordoma remains controversial, our results, among others, do not clearly support neoadjuvant radiation in chordoma (4, 39).

To our knowledge, ours is the first study evaluating the expression and prognostic role of cyclin E1 specifically in chordoma. While we have identified a significant correlation between cyclin E1 expression and chordoma patient prognosis, future works should focus on the mechanism of the overexpression of cyclin E1 in chordoma and how cyclin E1 overexpression causes chordoma cell proliferation and poor outcomes. This would inform future targeted therapies and clinical trials making use of our initial findings. Moreover, while molecular targeting therapies have been investigated in chordoma but have largely been underwhelming, the cyclin E1 should be investigated as a novel therapeutic strategy for chordoma patients (40–43).

There are some limitations in our study. First, 75 chordoma cases is comparatively large sample size for this rare disease as compared to other previous studies on investigating prognostic biomarkers in chordoma (7, 41, 42). However, future studies to increase the number of specimens are needed to validate cyclin E1 as a biomarker for chordoma prognosis. Second, no chordoma samples in our study were from skull base. It might be attributed to that all chordoma samples included in this study were surgically treated *via* orthopedic surgeons, who usually treated chordoma on mobile spine or sacrum. Third, the mechanisms of cyclin E1 overexpression in chordoma remains unknown and need be further investigated. Future investigations to validate and build on the prognostic value of Cyclin E1 in chordoma will be carried out on a larger sample size collaborating with neurosurgeons to include skull base chordoma, and will focus on the detail mechanism and regulation of cyclin E1 expression in chordoma.

## Conclusion

In summary, we show cyclin E1 is overexpressed in most chordomas, and its expression positively correlates to disease status and inversely correlates with OS, RFS, and MFS of chordoma patients. Our work suggests cyclin E1 as a prognostic biomarker for chordoma patients and supports future works investigating its mechanism and pathway as a potential drug target.

## Data Availability Statement

The raw data supporting the conclusions of this article will be made available by the authors, without undue reservation.

## Ethics Statement

The study protocol and the consent of the informed patients were approved by the Partners Human Research Committee (number: 2007-P-002464/5) of Partners HealthCare as previously described. All patients provided their written informed consent to participate in this study.

## Author Contributions

Research design and data preparation: RW, PT, FH, WG, and ZD. Drafting and revising the paper: RW, DD, and ZD. Approval of the submitted and final versions: WG and ZD. All authors contributed to the article and approved the submitted version.

## Funding

This work was supported, in part, by the Musculoskeletal Tumor Center and Beijing Key Laboratory of Musculoskeletal Tumor at Peking University People’s Hospital, Beijing, China, and Department of Orthopaedic Surgery at UCLA, Los Angeles, California, USA. WG is supported, in part, through a grant from Sanming Project of Medicine in Shenzhen, China. ZD is supported, in part, through a grant from Sarcoma Foundation of America (SFA), 222433, a Grant from National Cancer Institute (NCI)/National Institutes of Health (NIH), U01, CA151452-01.

## Conflict of Interest

The authors declare that the research was conducted in the absence of any commercial or financial relationships that could be construed as a potential conflict of interest.
